# Layered Double Hydroxide@Metal–Organic Framework Hybrids for Extraction of Indole-3-Carbinol From Cruciferous Vegetables

**DOI:** 10.3389/fnut.2022.841257

**Published:** 2022-05-17

**Authors:** Qiyue Tan, Guangyang Liu, Chenxi Zhao, Mingkun Gao, Xuan Zhang, Ge Chen, Lingyun Li, Xiaodong Huang, Yaowei Zhang, Jun Lv, Donghui Xu

**Affiliations:** ^1^Key Laboratory of Vegetables Quality and Safety Control, Laboratory of Quality and Safety Risk Assessment for Vegetable Products, Ministry of Agriculture and Rural Affairs of China, Institute of Vegetables and Flowers, Chinese Academy of Agricultural Sciences, Beijing, China; ^2^College of Horticulture, Northeast Agricultural University, Harbin, China; ^3^Hebei Key Laboratory of Quality & Safety Analysis-Testing for Agro-Products and Food, Hebei North University, Zhangjiakou, China

**Keywords:** indole-3-carbinol, metal-organic framework, layered double hydroxide, extraction, cruciferous vegetable

## Abstract

Cruciferous vegetables are rich in glucosinolates, which can be metabolized to produce the antitumor compound indole-3-carbinol (I3C). The conventional solvent extraction method for I3C is inefficient. To improve the extraction efficiency of I3C from cruciferous vegetables, we prepared a metal-organic framework (MOF) material (Fe_3_O_4_@Zn-Al-LDH@B-D-MIL-100). First, Fe_3_O_4_ nanoparticles were introduced to layered double hydroxides by *in situ* polymerization. Then, the MOF material was grown on the surface of the layered double hydroxide by co-precipitation and the layer-by-layer self-assembly method. This gave Fe_3_O_4_@Zn-Al-LDH@B-D-MIL-100, which was characterized using a variety of techniques. The results showed that Fe_3_O_4_@Zn-Al-LDH@B-D-MIL-100 had a double-layer porous structure, excellent superparamagnetism (11.54955 emu/g), a large specific surface area (174.04 m^2^/g), and a pore volume (0.26 cm^3^/g). The extraction conditions for I3C were optimized. Non-linear fitting of the static adsorption model showed that the adsorption was mainly monolayer. Fe_3_O_4_@Zn-Al-LDH@B-D-MIL-100 had fast adsorption kinetics and could extract 95% of I3C in 45 min. It is superior to the traditional solvent extraction method because of its high enrichment efficiency in a short time and environmental friendliness. The successful preparation of the new nanomaterial will provide a new reference for the enrichment and extraction of the I3C industry.

## Introduction

Cruciferous vegetables are rich in many vitamins, dietary fiber, and phytochemicals that are beneficial to human health. It has been widely reported that a moderate intake of cruciferous vegetables inhibits cancer development. The Japanese Center for Public Health reported a negative association between cruciferous vegetable intake and lung cancer risk in non-smoking men (1). A controlled study of hospital cases (2) found that cruciferous vegetable consumption reduced lung cancer risk, and another study (3) showed that cruciferous vegetable consumption potentially altered the risk of ovarian cancer. Glucosinolates are a class of glycosides found in the roots, stems, and fruits of cruciferous plants. Among them, 3-indole-methyl glucosinolate can be degraded by myrosinase or catalyzed by bacterial enzymes in the gastrointestinal tract (4) to produce indole-3-carbinol (I3C). I3C is recognized as a natural anticancer substance, which can induce apoptosis and inhibit tumorigenesis (5). Zhang et al. (6) found that the reduced risk of breast cancer associated with consumption of cruciferous vegetables was mainly due to I3C, which is produced by glucosinolate metabolism. Mohammadi et al. (7) found that I3C exerted its antileukemic effect by activating the aromatic hydrocarbon receptor, inducing programmed cell death. I3C is also of interest because it prevents prostate cancer (8), inhibition of *Citrobacter* infection to prevent colorectal cancer (9), and prevention of cerebral ischemia (10). The development of efficient extraction techniques for I3C is key to its further processing and use.

Presently, natural I3C is mainly extracted using traditional organic solvents, such as ethyl acetate (11) and dichloromethane (12), and then preserved with methanol or ethanol. Fusari et al. (13) investigated the extraction of I3C and other substances from cruciferous vegetables using liquid-liquid microextraction and optimized the extraction solvent, dispersion solvent, and other factors. The best extraction of I3C and other substances was obtained using 1 mL of acetonitrile as the dispersion solvent and 700 mL of chloroform as the extraction solvent. Suparman et al. (14) found that a 4:1 (v/v) mixture of DMF and methanol could effectively extract compounds such as I3C from freeze-dried vegetable samples. Although the organic solvent extraction method is capable of crude extraction of natural I3C, it has a low extraction efficiency, and the organic solvents used are toxic. Therefore, the development of a novel environmentally friendly extraction method is important. Garcia-Ibanez et al. (15) tried to improve the stability and retention of I3C by encapsulating aqueous extracts of red kale in plasma membrane vesicle nanomaterials.

In recent years, metal-organic frameworks (MOFs), a class of reticular, porous structured materials composed of metal ions and organic ligands (16), have been widely used in adsorption, catalysis, and medical imaging (17). These MOFs have large specific surface areas, high porosity, adjustable pore channels, and extremely high chemical and physical stability (18). MOFs are promising in the field of adsorption. Liu et al. (19) prepared a novel magnetic copper-based MOF using Fe_3_O_4_–graphene oxide–β-cyclodextrin as a magnetic core and carrier, and applied for the adsorption of neonicotinoid pesticide contaminants. Li et al. (20) prepared a novel MOF (M-ZIF-8@ZIF-67) for the adsorption of fipronil and its metabolites. Hu et al. (21) prepared a composite material (CGUNCM) by attaching chitosan to UiO-66-NH_2_ and used it for adsorption of the heavy metals Cu (II) and Pb (II) in aqueous solutions. In addition to the adsorption of pollutants such as heavy metals (22) and pesticides (23), MOFs have been applied to extract bioactive substances. For example, Mensinger et al. (24) synthesized seven MOFs and investigated their different adsorption rates and retention strengths for amyloid β-peptides. Rupa et al. (25) prepared *Dendropanax morbifera* zinc oxide nanoparticles using a co-precipitation method and loaded I3C into the nanomaterials using an ultrasonic technique, which allowed for later release of I3C. Although the simple three-dimensional MOF structure has a very high specific surface area and molecular loading, it is not easily dispersed in an aqueous solution, which limits the accessibility of target molecules to active sites (26). If nanocarriers are introduced to transform the three-dimensional MOFs structure into a two-dimensional one, the resulting material is ultrathin and has a larger specific surface area and more active sites than the original MOF (27). Layered double hydroxides (LDH) are two-dimensional layered structures that are easy to synthesize and can provide binding sites for polymers to form composites (28). LDH can be adsorbed by both surface adsorption and ion exchange (29). Chen et al. (30) designed a defective metal-organic backbone (B-D-MIL-100) nanoreactor modified with boric acid to improve the separation efficiency of a MOF. Because Fe_3_O_4_ has excellent magnetic properties, the addition of Fe_3_O_4_ to the LDH surface and combination with a three-dimensional MOF to form a two-dimensional structure should in theory improve the material loading rate greatly.

In this study, a novel MOF nanomaterial (Fe_3_O_4_@Zn-Al-LDH@B-D-MIL-100) was successfully prepared by homogeneous co-precipitation, *in situ* polymerization, and layer-by-layer self-assembly. Quantitative detection by high-performance liquid chromatography with a tandem triple quadrupole mass spectrometer (HPLC-MS/MS) was used to optimize the concentration, mass, time, temperature, pH, and ionic strength. Efficient extraction of I3C was successfully achieved. The adsorption materials used for the enrichment of I3C are low-cost and environmentally friendly, and the use of toxic organic solvents is avoided in the enrichment process. This enrichment method is expected to provide a reference for the development and utilization of more I3C products.

## Materials and Methods

### Chemicals and Reagents

Anhydrous sodium carbonate and zinc nitrate were purchased from Tianjin Fuchen Chemical Reagent Co. (Tianjin, China). 3,5-Dicarboxyphenylboronic and trimesic acid were obtained from Beijing Huawei Si Ke Technology Co. (Beijing, China). Methanol and Aluminum nitrate nonahydrate were purchased from Sinopharm Chemical Reagent Co. (Shanghai, China). Sodium hydroxide was sourced from Beijing Beihua Fine Chemicals Co. (Beijing, China). Ammonium hydroxide was obtained from Sigma-Aldrich Trading Co. (Shanghai, China). Iron chloride hexahydrate, Ferrous sulfate heptahydrate, and indole-3-carbinol were purchased from Shanghai Maclean Co. (Shanghai, China).

### Instruments and Equipment

Scanning electron microscopy (SM-6300, JEOL, Tokyo, Japan) was used to characterize the particle size and morphology of Zn-Al-LDH, B-D-MIL-100, Fe_3_O_4_@Zn-Al-LDH, and Fe_3_O_4_@Zn-Al-LDH@ B-D-MIL-100. The particle size, morphology, and properties of Fe_3_O_4_ were characterized by a transmission electron microscope (JEM-200CX, JEOL, Tokyo, Japan). The Brunauer-Emmett-Teller (BET) surface area and N_2_ adsorption and desorption isotherms of Fe_3_O_4_@Zn-Al-LDH@B-D-MIL-100 were measured by the ASAP 2020 analyzer (Micromeritics Instrument Corp., Norcross, GA, United States). The crystal structure of all materials was analyzed using an advanced X-ray powder diffractometer (Bruker AXS GmbH, Karlsruhe, Germany). Fourier transform infrared spectra of all synthesized nanomaterials and I3C adsorbents were recorded using a Fourier spectrophotometer (Nicolet 6700, Thermo Fisher Scientific, Madison, WI, United States). X-ray photoelectron spectrograms of all nanomaterials were tested using an X-ray photoelectron spectrometer (EscaLab 250Xi, Thermo Fisher Scientific, WI, United States). Raman spectrograms of all synthesized nanomaterials and I3C adsorbents were analyzed using a Raman spectrometer (Renishaw inVia, Renishaw, United Kingdom). Magnetic properties of Fe_3_O_4_, Fe_3_O_4_@Zn-Al-LDH, and Fe_3_O_4_@Zn-Al-LDH@ B-D-MIL-100 nanomaterials were determined by vibrating sample magnetometry (Lake Shore 7410, United States). I3C was quantified by a high-performance liquid chromatography-tandem mass spectrometer (LC/MSMS-8050; Shimadzu, Japan).

### Synthesis of Zn-Al-LDH

First, 50 mL of deionized water containing 4 mM Zn^2+^ and 2 mM Al^3+^ was slowly added to 100 mL of 0.1 mol/L Na_2_CO_3_ solutions with magnetic stirring for 2 h. The pH of the solution was adjusted to 10 with 2 mol/L NaOH and stirring was continued at room temperature for 2 h. The solution was then centrifuged at 8,000 rpm for 10 min in a high-speed refrigerated centrifuge (3K15, Beijing Tianlin Hengtai Technology Co.), the supernatant was decanted, and the white precipitate was collected. The precipitate was washed twice with water and anhydrous ethanol sequentially, freeze-dried for 12 h, and then ground to obtain a white powder.

### Synthesis of Fe_3_O_4_@Zn-Al-LDH

Deionized water (240 mL) and Zn-Al-LDH (0.2 g) were added to a 500 mL three-necked flask. Another 20 mL of deionized water was placed in a 50 mL centrifuge tube with 0.35 g of FeSO_4_-7H_2_O and 0.6 g of FeCl_3_-6H_2_O. The tube was vortex mixed until the solids dissolved. The solution was then filtered into the three-necked flask through a 0.22-μm filter membrane. The mixture in the flask was stirred magnetically at 80°C for 30 min. Next, 10 mL of ammonia solution (28% purity) was added and stirring was continued at 80°C for 30 min. After stirring, the mixture was cooled to room temperature. Magnetic separation was performed, and the collected material was washed twice with anhydrous ethanol and water sequentially, freeze-dried for 12 h, and ground to obtain a black-brown powder.

### Synthesis of Fe_3_O_4_@Zn-Al-LDH@B-D-MIL-100

**FIGURE 1:**
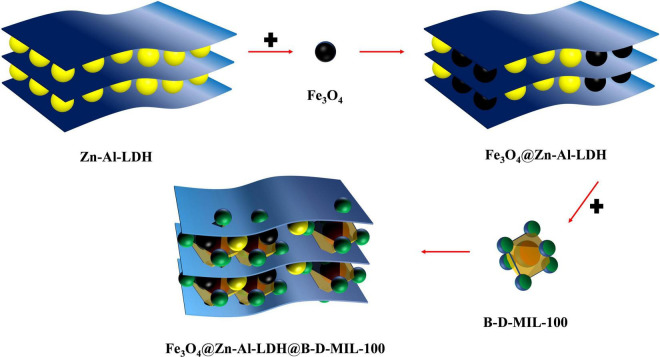
Process for the synthesis of composite materials.

The Fe_3_O_4_@Zn-Al-LDH (0.35 g) synthesized in section “Synthesis of Fe3O4@Zn-Al-LDH” was dispersed in 5 mL of an ethanol solution containing 0.027 g of FeCl_3_-6H_2_O. This mixture was then heated at 70°C for 15 min and centrifuged at 8,000 rpm for 10 min. The precipitate was collected and washed three times with ethanol. Next, 10 mL of an ethanol solution containing 0.0126 g of 1,3,5-benzene tricarboxylic acid and 0.0085 g of 3,5-dicarboxybenzeneboronic acid was added, and the mixture was heated at 70°C for 30 min. After centrifugation, the precipitate was collected and washed three times with ethanol to obtain the material precursor. An ethanol solution of the material precursor was prepared and 60 mL of this was added to a three-neck flask with 0.270 g of FeCl_3_-6H_2_O, 0.1260 g of 1,3,5-benzene tricarboxylic acid, and 0.0851 g of 3,5-dicarboxybenzeneboronic acid. The mixture was stirred magnetically at 70°C for 12 h. The product was isolated by magnetic separation, washed three times with ethanol, freeze-dried for 12 h, and ground to obtain a brown powder (31) (Figure 1).

### Standard Solution Preparation and Sample Pretreatment

I3C (1.0 g) was dissolved in a 100 mL volumetric flask with methanol and the volume was made up to the mark to obtain a 1,000 μg/mL I3C standard solution. The standard solution was stored in a refrigerator at 4°C and diluted as required for use.

In July 2021, three cruciferous vegetables, including broccoli, kale, and collards, were ordered from The Daily Premium Store (Lenovo Bridge store, Haidian District, Beijing). The broccoli stems were removed and the heads were cut into 1-cm pieces. Kale and collard greens were cut into 1-cm slices. The cut vegetable samples were placed in sample bags in a refrigerator at −20°C for 2 h. The pre-chilled samples were then freeze-dried for 3 d, ground to powders, and set aside.

Powdered samples of broccoli, kale, and cabbage were weighed (0.5 g each) into 50 mL centrifuge tubes. PBS buffer (0.1 M, 15 mL) was added and the tubes were shaken for 90 min on a speed-controlled multipurpose shaker (32). Ethyl acetate (30 mL) was then added to each tube for I3C extraction, and the tubes were shaken for 30 min. After centrifugation at 8,000 rpm for 10 min, the upper soluble layer of ethyl acetate was moved to a round-bottom flask. After three cycles of extraction, the ethyl acetate was evaporated at 30°C under vacuum, and the residue was dissolved in 10 mL of methanol. The extract was filtered through a 0.22 μm filter membrane and packed into a 2 mL brown vial for analysis by HPLC-MS/MS. For extraction by the composite, 10 mL centrifuge tubes containing Fe_3_O_4_@Zn-Al-LDH@B-D-MIL-100 samples (20, 30, and 50 mg) were prepared in triplicate. The three vegetable powder samples were then weighed (0.5 g) into separate 10 mL centrifuge tube with PBS buffer (0.1 M, 15 mL), shaken for 90 min on a speed-controlled multipurpose shaker, and then centrifuged for 10 min at 8,000 rpm. The supernatant from each sample was removed and aliquots were added to the tubes containing the nanocomposite (20, 30, and 50 mg). These tubes were shaken for 45 min, and then magnetic separation was performed. The supernatant was centrifuged for 10 min, and then filtered through a 0.22 μm filter membrane into a 2 mL brown vial, diluted, and analyzed.

### Adsorption Evaluation

The amount adsorbed, *Q*, was calculated using the following equation:


(1) $$Q=\frac{\left(C_{0}-C_{c}\right)}{m}\cdot V,$$

The extraction efficiency was calculated to evaluate the adsorption performance of the nanocomposites for I3C as follows:


(2) $$\mathrm{Extraction}\,\mathrm{efficiency}=\frac{\left(C_{0}-C_{t}\right)}{C\,0}\times 100\%,$$

where *C*_0_ (μg/mL) is the concentration of the I3C solution before adsorption, *C*_*c*_ (μg/mL) is the concentration of the solution after adsorption on the nanocomposite, *V* (mL) is the volume of the aqueous I3C solution, *m* (mg) is the mass of the nanocomposite, and *C*_*t*_ (μg/mL) is the concentration of I3C at the adsorption equilibrium.

The Langmuir and Freundlich isotherm adsorption models (Eqs 3 and 4) were used to evaluate the adsorption process of I3C on the nanocomposite and the adsorption performance of the nanocomposite at equilibrium.


(3) $$\frac{C_{e}}{q_{e}}=\frac{1}{K_{L}\,q_{m}}+\frac{C_{e}}{q_{m}},$$

(4) $$\ln q_{e}=\ln K_{F}+\frac{1}{n}\,\ln C_{e},$$

where *q*_*e*_ (mg/g) is the mass of I3C adsorbed on the composite material at adsorption equilibrium, *q*_*m*_ (mg/g) is the maximum mass of I3C adsorbed by the composite material, *C*_*e*_ (μg/mL) is the concentration of I3C at equilibrium, *K*_*L*_ is the Langmuir constant, *K*_*F*_ is the Freundlich constant, and *1/n* is the adsorption index.

### High-Performance Liquid Chromatography With a Tandem Triple Quadrupole Mass Spectrometer

Phenomenex Kinetex C_18_ column (50 mm × 3 mm, 2.6 μm) was used in the experiment, the column temperature was 40°C, and the detection wavelength was 280 nm. 5μL was injected each time at the flow rate of 0.3 mL/min. Methanol was used as mobile phase A and 1.0 m mol/L ammonium acetate as mobile phase B. Multiple Reaction Monitoring (MRM), negative ion scanning, and electrospray ion source (ESI) were used. The parent ion m/z of I3C is 146.0, the impact energy is 17 eV, the interface voltage is 4.0 kV, the interface temperature is 300°C, the dissolvent temperature is 250°C, and the helium drying flow is 10 L/min.

### Data Analysis

The characterization data of X-ray diffraction technology and X-ray photoelectron spectroscopy test were processed by MDI Jade software and Avantage software, respectively. Then Origin data analysis software was used to analyze and process the adsorption optimization experimental data and other characterization data, and finally, the analysis graph was made.

## Results

### Material Characterization

The particle sizes and morphologies of Fe_3_O_4_, Zn-Al-LDH, B-D-MIL-100, Fe_3_O_4_@Zn-Al-LDH, and Fe_3_O_4_@Zn-Al-LDH@B-D-MIL-100 were determined by scanning electron microscopy and transmission electron microscopy.

**FIGURE 2:**
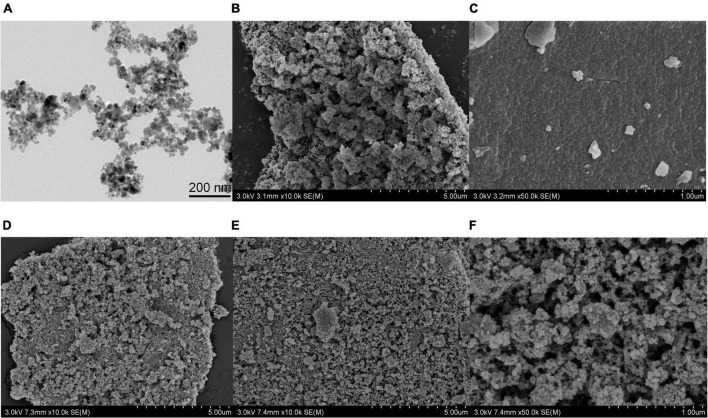
**(A)** TEM image of Fe_3_O_4_. SEM images of **(B)** Zn-Al-LDH, **(C)** B-D-MIL-100, **(D)** Fe_3_O_4_@Zn-Al-LDH, **(E,F)** Fe_3_O_4_@Zn-Al-LDH@B-D-MIL-100.

The Fe_3_O_4_ nanoparticles had a dispersed spherical structure (Figure 2A), which would be favorable for uniform dispersion on the surface of the Zn-Al-LDH material with layered porous structure (Figure 2B). The Fe_3_O_4_ was successfully dispersed on the surface of Zn-Al-LDH to form Fe_3_O_4_@Zn-Al-LDH (Figure 2D). Figure 2C is the structure diagram of nanomaterials B-D-MIL-100, and Figure 2D is the morphology structure of Fe_3_O_4_@Zn-Al-LDH formed by the successful dispersion of Fe_3_O_4_ on the surface of Zn-Al-LDH. Combining the structural characteristics of B-D-MIL-100 and Fe_3_O_4_@Zn-Al-LDH, it can be seen that Fe_3_O_4_@Zn-Al-LDH successfully grows on the surface of B-D-MIL-100 (Figures 2E,F). The Fe_3_O_4_@Zn-Al-LDH@B-D-MIL-100 had a good pore size (Figure 2F) and coupled with its magnetic properties, this would be very favorable for use in extraction.

**FIGURE 3:**
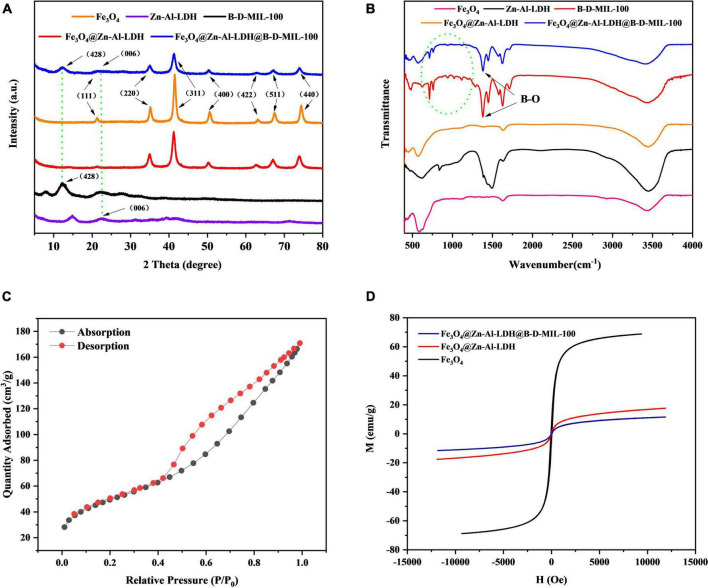
**(A)** X-ray diffraction, **(B)** FT-IR, **(C)** BET, and **(D)** magnetic characterization of the composites.

The crystal structures of Fe_3_O_4_, Zn-Al-LDH, B-D-MIL-100, Fe_3_O_4_@Zn-Al-LDH, and Fe_3_O_4_@Zn-Al-LDH@B-D-MIL-100 were characterized by X-ray diffraction. All five materials showed characteristic diffraction peaks (Figure 3A). Fe_3_O_4_@Zn-Al-LDH@B-D-MIL-100 had nine diffraction peaks. Comparison with a standard showed that the diffraction peaks at 2θ 21.304°, 35.048°, 41.325°, 50.423°, 63.132°, 67.223°, and 74.123° were associated with the (111), (220), (311), (400), (422), (511), and (440) crystallographic positions, respectively. The diffraction peak located at 2θ 21.622°corresponded to the (006) crystallographic position of Zn-Al-LDH. The diffraction peak located at 2θ 12.309°corresponded to the (428) crystallographic position of B-D-MIL-100. These results show that Fe_3_O_4_, Zn-Al-LDH, and B-D-MIL-100 were successfully incorporated into Fe_3_O_4_@Zn-Al-LDH@B-D-MIL-100.

FT-IR spectra of Fe_3_O_4_, Zn-Al-LDH, B-D-MIL-100, Fe_3_O_4_@Zn-Al-LDH, and Fe_3_O_4_@Zn-Al-LDH@B-D-MIL-100 were recorded from 480 to 4,000 cm^–1^ (Figure 3B). A peak for Fe-O was observed at 580 cm^–1^, which indicated that Fe_3_O_4_ was successfully incorporated into the composite. Overlapping peaks for B-D-MIL-100 and Fe_3_O_4_@Zn-Al-LDH@B-D-MIL-100 occurred at 1323.92 cm^–1^, which indicated that many boron-oxygen bonds were present in the composite. These bonds would be conducive to chemical bonding with the target, which would enhance the adsorption performance of the material.

The N_2_ adsorption-desorption isotherm of Fe_3_O_4_@Zn-Al-LDH@B-D-MIL-100 (Figure 3C) was consistent with a type III isotherm. At low pressures, the composite adsorbed less, and at higher pressures, the material provided rapid adsorption. These results indicated that the composites had mesoporous structures and high-pressure adsorption was the main adsorption pathway. The pore size and surface area of Fe_3_O_4_@Zn-Al-LDH@B-D-MIL-100 were determined. The Brunauer-Emmett-Teller (BET) specific surface area of the composite was 174.04 m^2^/g. The average pore diameter and pore volumes were 6.00166 nm and 0.261132 cm^3^/g, respectively. The large specific surface area and structurally stable pore structure will be conducive to effective adsorption.

Hysteresis curves were obtained for Fe_3_O_4_, Fe_3_O_4_@Zn-Al-LDH, and Fe_3_O_4_@Zn-Al-LDH@B-D-MIL-100 (Figure 3D), and showed that the three materials had good superparamagnetism. Compared with Fe_3_O_4_, the magnetic properties of the other two materials were lower, but they still had good magnetic separation ability. The magnetization strengths of Fe_3_O_4_, Fe_3_O_4_@Zn-Al-LDH, and Fe_3_O_4_@Zn-Al-LDH@B-D-MIL-100 at room temperature were 68.71, 17.50, and 11.51 emu/g, respectively.

**FIGURE 4:**
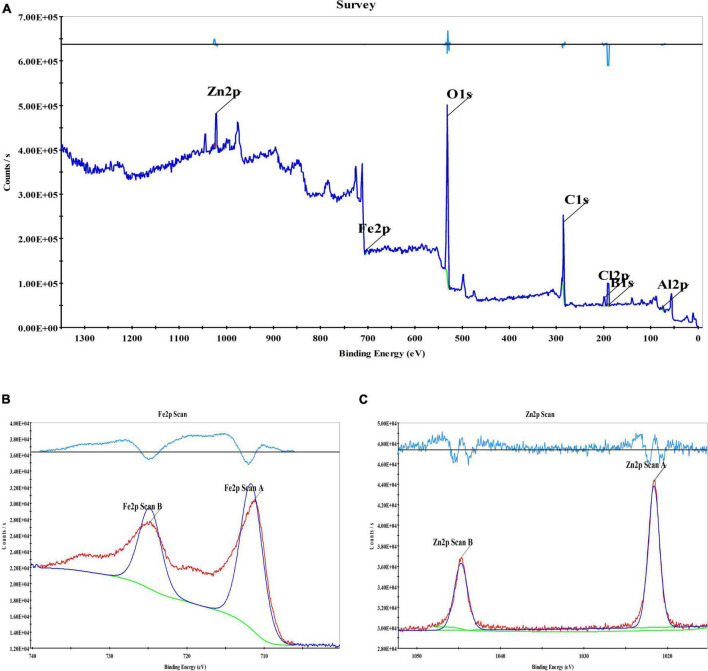
XPS spectrogram of the composite materials **(A)**, XPS spectrogram of Fe **(B)**, and XPS spectrogram of Zn **(C)**.

X-ray photoelectron spectroscopy was performed on Fe_3_O_4_@Zn-Al-LDH@B-D-MIL-100 (Figure 4). The spectrum showed that the material contained Zn, Fe, B, Al, O, and other important elements. Analysis of the sub-spectra for the elements showed that both Fe and Zn had two valence states (divalent and trivalent). Overall, the elemental analysis showed that Fe_3_O_4_@Zn-Al-LDH@B-D-MIL-100 was successfully synthesized.

**FIGURE 5:**
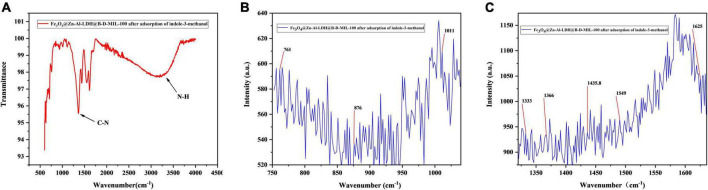
**(A)** FT-IR, and **(B,C)** Raman spectra of composite with extracted I3C.

After the adsorption of I3C, the Fe_3_O_4_@Zn-Al-LDH@B-D-MIL-100 was characterized by FT-IR and Raman spectroscopy (Figure 5). Comparison of the FT-IR spectrum (Figure 5A) with other results (33, 34) showed that it contained a peak for N-H on the indole ring at 3,314 cm^–1^ and C-N on the indole ring at 1,361 cm^–1^. The Raman spectrum (Figures 5B,C) contained peaks for the indole ring at 761, 876, 1,011, 1,333, 1435.8, 1,366, 1,549, and 1,625 cm^–1^, and N-H on the indole ring at 1435.8 cm^–1^ (35). These results indicated that I3C was successfully extracted by Fe_3_O_4_@Zn-Al-LDH@B-D-MIL-100.

### Optimization of the Adsorption Performance

#### Material for Extraction

**FIGURE 6:**
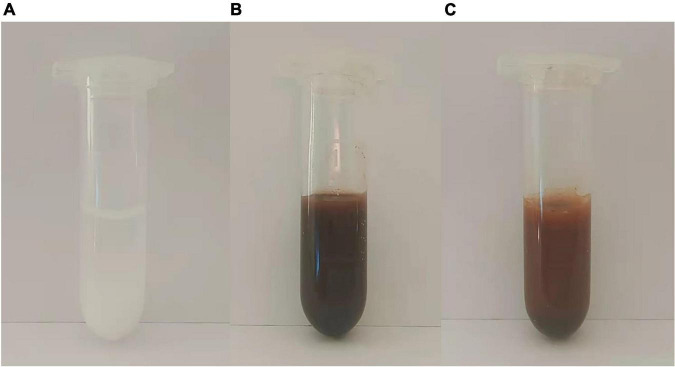
Photographs of **(A)** Zn-Al-LDH, **(B)** Fe_3_O_4_@Zn-Al-LDH, and **(C)** Fe_3_O_4_@Zn-Al-LDH@B-D-MIL-100.

To provide the same molar mass of Zn, we used 2 mg of Zn-Al-LDH (Figure 6A), 8.655 mg of Fe_3_O_4_@Zn-Al-LDH (Figure 6B), and 8.728 mg of Fe_3_O_4_@Zn-Al-LDH@B-D-MIL-100 to extract I3C from a 100 μg/mL solution (Figure 6C) aqueous solution. The supernatant was analyzed after 10 min of contact between each material and the I3C aqueous solution. The extraction efficiencies of Zn-Al-LDH, Fe_3_O_4_@Zn-Al-LDH, and Fe_3_O_4_@Zn-Al-LDH@B-D-MIL-100 for I3C were 16.75, 25.90, and 93.19%, respectively. These results showed that Fe_3_O_4_@Zn-Al-LDH@B-D-MIL-100 had the best extraction efficiency among the tested materials.

#### Concentration

**FIGURE 7:**
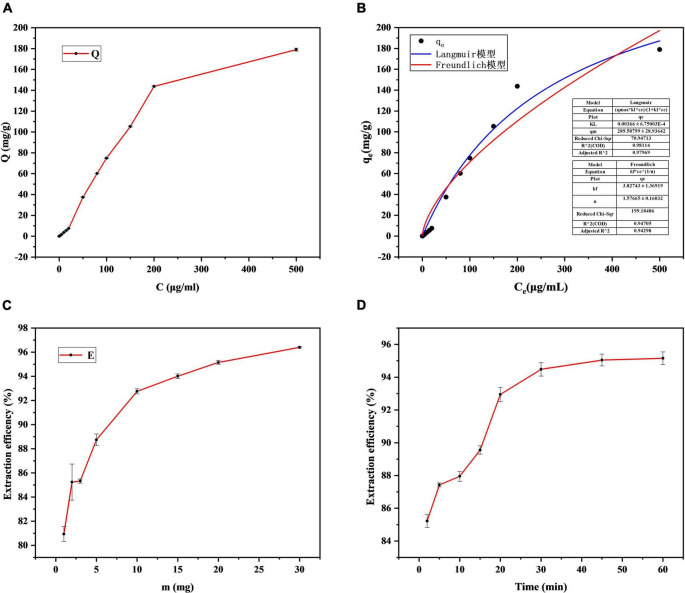
Optimization of the **(A)** concentration, **(C)** mass, and **(D)** contact time, and **(B)** non-linear fitting of Langmuir and Freundlich models.

Standard solution of I3C diluted with deionized water (500, 200, 150, 100, 80, 50, 20, 15, 10, 5, 2, 1, 0.5, 0.2, 0.1 μg/mL) into centrifugal tubes containing 10 mg Fe_3_O_4_@Zn-Al-LDH@B-D-MIL-100 composite material. Shaken at room temperature for 30 min, magnetic separation. The concentration of I3C in the supernatant was determined by HPLC-MS/MS, and the relationship between adsorption mass and concentration was plotted (Figure 7A). The adsorption mass of Fe_3_O_4_@Zn-Al-LDH@B-D-MIL-100 increased gradually with increases in the concentration of the I3C solution. Therefore, the composite material performs well as an adsorbent and could be used for the extraction of I3C. To further optimize the extraction conditions, we selected 100 μg/mL I3C as the optimum concentration.

Non-linear fitting of the Langmuir and Freundlich models for the extraction of I3C at different concentrations was performed using Origin software (Figure 7B). The goodness of fit *R*^2^ of the Langmuir model for I3C adsorption data reached 0.98%, and the mean value of q_*m*_ was 289.58 mg/g. The goodness of fit *R*^2^ of the Freundlich model was also high, reaching 0.94, and the mean value of K_*F*_ was 3.82. In comparison, the Langmuir model has a higher degree of consistency, and monolayer adsorption mainly occurs on materials. According to the previous studies by Chen et al. (36), the physicochemical properties of adsorption reactions can be judged by the size of the adsorption constant n in the Freundlich model. n (1.57 ± 0.16) > 1 of the adsorption constants fitted in this study indicates that the adsorption of I3C by Fe_3_O_4_@Zn-Al-LDH@B-D-MIL-100 is mainly a physical process.

#### Mass of the Composite

A total of 4 mL I3C (100 μg/mL) standard solution was added into the centrifuge tube containing Fe_3_O_4_@Zn-Al-LDH@B-D-MIL-100 (2, 3, 5, 10, 15, 20, or 30 mg) composite material. After shaking the tubes for 30 min at room temperature, the concentration of I3C was quantified using HPLC-MS/MS. A graph of the extraction efficiency against the mass of Fe_3_O_4_@Zn-Al-LDH@B-D-MIL-100 showed that the maximum extraction efficiency (95.15%) was reached with 20 mg of the adsorbent (Figure 7C). Therefore, 20 mg was selected as the optimum dose for subsequent experiments.

#### Contact Time

The contact time between the adsorbent and the target affects the extraction efficiency. To find the optimum contact time, a mixture of the composite (20 mg) and I3C (100 μg/mL) was shaken for 2, 5, 10, 15, 20, 30, 45, or 60 min before magnetic separation and quantification. The extraction efficiency increased with increases in the contact time (Figure 7D) and tends to be stable after 45 min, Therefore, 45 min was selected as the optimum contact time.

#### pH Optimization

**FIGURE 8:**
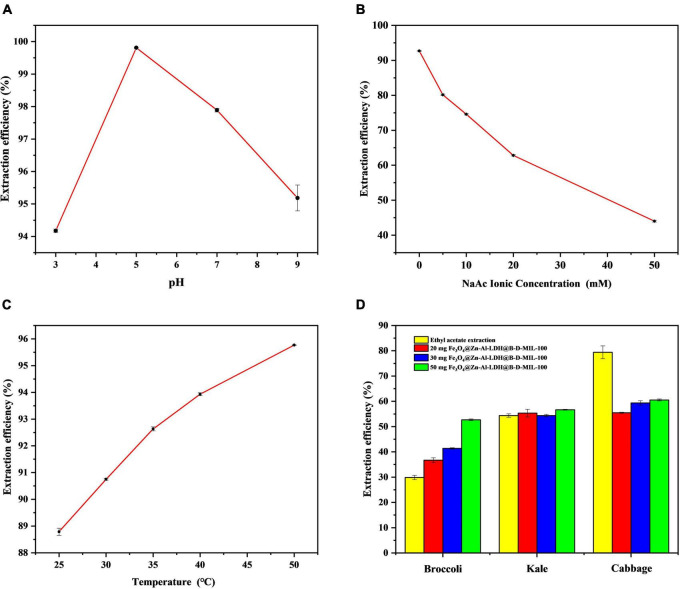
Optimization of the **(A)** pH, **(B)** ionic strength, and **(C)** temperature, and **(D)** actual sample extraction results.

The pH was optimized using values of 3, 5, 7, and 9 (Figure 8A). The best extraction of I3C from a 100 μg/mL aqueous solution onto 20 mg of the composite material was achieved at pH 5. This pH value gave an extraction efficiency of 99.81% with a relative standard deviation (RSD) of 0.0283%. The pH of the deionized water used in the laboratory was 5.8. Because this pH was close to the optimum pH, we used the deionized water in our experiments without adjusting the pH.

#### Ionic Strength

The ionic strength can affect the solubility and distribution of I3C in solution. To investigate the effect of ionic strength, we added sodium acetate (CH_3_COONa) to the 100 μg/mL standard solution of I3C at 5, 10, 20, and 50 mM. The concentration of I3C in the solution was determined by HPLC-MS/MS, and the extraction efficiency was plotted against the CH_3_COONa concentration (Figure 8B). As the concentration of CH_3_COONa increases, the adsorption efficiency of I3C becomes lower and lower, indicating that the synthesized material is limited by a certain ionic strength. However, when the ion concentration is low, the material is relatively stable and the adsorption efficiency can be more than 70%. Consequently, subsequent experiments were conducted without the addition of CH_3_COONa.

#### Temperature Optimization

We investigated the effect of temperature (25, 30, 35, 40, and 50°C) on the extraction efficiency of I3C. The extraction efficiency increased with increases in the temperature (Figure 8C). However, the increase in temperature also led to partial degradation of I3C. The higher the temperature, the more I3C degradation. For example, when the temperature reached 50°C, nearly half of the I3C was degraded. Therefore, we selected room temperature as the optimum temperature.

### Extraction of Indole-3-Carbinol From Actual Samples

The masses of I3C extracted from the broccoli, cabbage, and kale (0.5 g of each sample) after enzymatic digestion were 20.44, 4.53, and 5.24 μg, respectively. With ethyl acetate, 29.87, 54.38, and 79.42% of I3C could be extracted from broccoli, kale, and cabbage powder, respectively. The corresponding RSDs were 2.88, 1.26, and 3.21%. However, using 50 mg of Fe_3_O_4_@Zn-Al-LDH@B-D-MIL-100, the extraction efficiencies for I3C from broccoli, kale, and cabbage were 52.70, 56.68, and 60.54%, respectively. The corresponding RSDs were 0.62, 0.29, and 0.63% (Figure 8D). These results show that Fe_3_O_4_@Zn-Al-LDH@B-D-MIL-100 provided better extraction of I3C than the conventional ethyl acetate method for broccoli and kale. However, the extraction of I3C from cabbage using Fe_3_O_4_@Zn-Al-LDH@B-D-MIL-100 was relatively poor. Possible reasons for this are that the amount of the composite was insufficient or there were substances in cabbage that were more readily absorbed by the composite than I3C. It would be better if the samples were crude extracted before natural I3C enrichment with the composite Fe_3_O_4_@Zn-Al-LDH@B-D-MIL-100. Compared with previous extraction methods, the extraction time of this study is shorter and the efficient extraction of I3C can be achieved within 45 min, without the use of toxic organic solvents. Several extraction methods for I3C are compared in Table 1.

**TABLE 1:** Comparison of different extraction methods for indole-3-carbinol.

Extraction method	Extracting time	Testing instrument	Extraction efficiency	Experimental sample	Organic solvent	References
Molecularly imprinted polymer-Solid-phase extraction (MIP-SPE)	16 h	UV–vis spectrophotometer	95%	The mixed standard containing I3C, indole-3-acetonitrile, teophylline, and tryptophan	–	(37)
Liquid-liquid extraction	3 h	UPLC–HRMS/MS	–	Broccoli	Dichloromethane	(12)
Liquid-liquid extraction	2 h	HPLC	99.25%	Cabbage, Broccoli	Ethyl acetate	(38)
Solid-phase extraction	–	HPLC-DAD-FLD	94.5%	Seeds of Brassica plants, Brussels sprouts, savoy cabbage	–	(32)
QuEChERS method Fe_3_O_4_@Zn-Al-LDH@B-D-MIL-100 extraction	20 min 45 min	UHPLC-MS/MS HPLC-MS/MS	97.3% 95%	Rapeseeds Broccoli, Kale, Cabbage	Dichloromethane –	(39) This work

## Conclusion

Fe_3_O_4_@Zn-Al-LDH@B-D-MIL-100 was prepared by *in situ* polymerizations, chemical co-precipitation, and layer-by-layer self-assembly. This material could rapidly and efficiently extract I3C. Magnetic nanocomposites with the double-layered porous structure are more easily dispersed in an aqueous solution and can provide more active sites for I3C compared with materials with a common three-dimensional structure, which greatly improves the enrichment efficiency. The extraction efficiency of 95% was obtained by shaking 20 mg of Fe_3_O_4_@Zn-Al-LDH@B-D-MIL-100 with 4 mL of I3C 100 μg/mL for 45 min. The magnetic separation process of composites is simple and the enrichment time is greatly saved. Compared with the traditional organic solvent extraction method, it also avoids the use of ethyl acetate, dichloromethane, and other organic solvents, the whole process is non-toxic, more friendly to the environment. Fe_3_O_4_@Zn-Al-LDH@B-D-MIL-100 was successfully applied to efficient extraction of I3C from several cruciferous vegetables. This method provides a new direction for the extraction and processing of I3C products in the future.

## Data Availability Statement

The raw data supporting the conclusions of this article will be made available by the authors, without undue reservation.

## Author Contributions

QT: conducting experiments, writing – original draft, and modifying the manuscript. GL, YZ, and DX: providing ideas, supervision, project administration, and funding acquisition. CZ, MG, and XZ: software, investigation and analyzing data. GC, LL, XH, and JL: modifying statement syntax and providing experimental instruments and methods.

## Conflict of Interest

The authors declare that the research was conducted in the absence of any commercial or financial relationships that could be construed as a potential conflict of interest.

## Publisher’s Note

All claims expressed in this article are solely those of the authors and do not necessarily represent those of their affiliated organizations, or those of the publisher, the editors and the reviewers. Any product that may be evaluated in this article, or claim that may be made by its manufacturer, is not guaranteed or endorsed by the publisher.
